# Qualitative Assessment of One Health Operationalization at the Central Level and in Two Border Districts of Sierra Leone

**DOI:** 10.1002/puh2.70338

**Published:** 2026-08-12

**Authors:** Joseph Anderson Bunting‐Graden, Fatmata Sawaneh, Alhassan Mayei, Monya Konneh, Patrick Fatoma, Emmanuel Komba Finoh, Lily Kainwo, Wesen Konteh, Mukeh Kenneth Fahnbulleh, Klaere Heyden, Mohamed Alex Vandi, Foday Sahr, Adel Hussein Elduma Abdalla, Rashid Ansumana, Kinley Wangdi, Philip P. Mshelbwala

**Affiliations:** ^1^ National Public Health Agency Freetown Sierra Leone; ^2^ Department of Biostatistics School of Public Health Njala University Bo Sierra Leone; ^3^ West African One Health Njala University Bo Sierra Leone; ^4^ Regional Programme Support to Pandemic Prevention in the ECOWAS Region (RPPP) Deutsche Gesellschaft für Internationale Zusammenarbeit (GIZ) GmbH Freetown Sierra Leone; ^5^ Sierra Leone Field Epidemiology Training Program (SLFETP) African Field Epidemiology Network (AFENET) Freetown Sierra Leone; ^6^ College of Medical Sciences Njala University Bo Sierra Leone; ^7^ School of Public Health and Tropical Medicine Tulane University New Orleans Louisiana USA; ^8^ HEAL Global Health Centre Health Research Institute Faculty of Health University of Canberra Bruce Australian Capital Territory Australia; ^9^ National Centre for Epidemiology and Population Health ANU College of Law Governance and Policy The Australian National University Acton Australian Capital Territory Australia; ^10^ Department of Primary Industries and Regional Development Orange New South Wales Australia; ^11^ School of Veterinary Science The University of Queensland Gatton Queensland Australia

**Keywords:** central, collaboration, coordination, Kailahun, Kambia, One Health, Zoonotic disease

## Abstract

Zoonotic diseases with epidemic potential pose a serious public health threat in Sierra Leone, where the One Health (OH) approach has been adopted to strengthen multi‐sectoral preparedness and response. This study assessed the operationalization of OH in responding to such diseases at the central level and in two districts, Kambia and Kailahun, using content analysis of semi‐structured interviews, focus group discussions and key documents. Manifest and latent analysis generated themes, including coordination and collaboration, surveillance and reporting, human resource capacity, communication, funding, and community engagement and awareness. Our study found that at the central level, OH sectors showed strong coordination, a functional surveillance system, established communication platforms and relatively strong capacity. District and community levels, by contrast, showed weak coordination, fragmented surveillance and limited communication, along with low animal health (AH) workforce capacity and OH awareness. Community level AH workers lacked an integrated approach to preventing, detecting and reporting zoonotic disease risks. Across all levels, OH activities lacked dedicated funding and relied solely on partner support, with the AH sector facing notable workforce shortages. OH, implementation was markedly weaker at district and community levels than at the central level, a disparity largely attributable to the absence of dedicated funding. Despite these gaps, the OH approach holds considerable potential for mitigating and managing zoonotic disease risks in Sierra Leone.

## Graphical abstract

Implementation of the One Health approach in Sierra Leone was stronger at the central level than in districts and communities, where coordination, surveillance, workforce capacity, and funding were limited. Strengthening integrated systems and dedicated financing is essential for effective prevention and control of zoonotic disease threats.

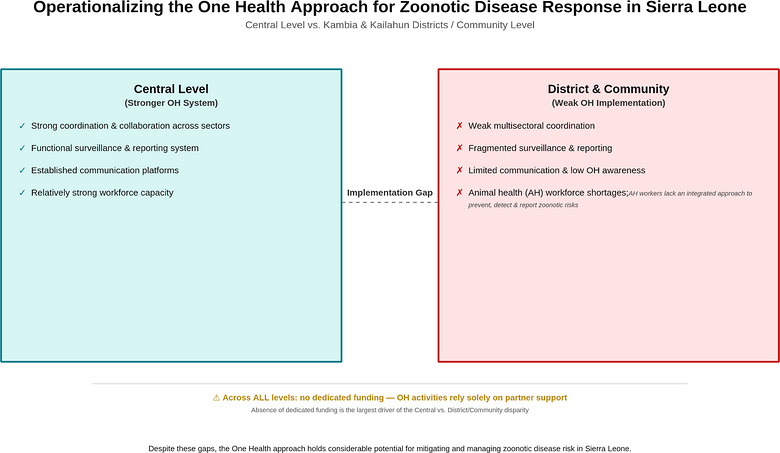

## 1 Background

Public health challenges at the human–animal–environment interface are becoming increasingly complex and dynamic, necessitating whole‐of‐society solutions [1, 2]. The One Health (OH) approach recognises that human, animal and environmental health are interconnected. By bringing together multiple sectors and disciplines, it aims to improve outcomes across all three [2]. It is particularly vital in addressing zoonotic disease threats, considering all components, including wildlife, as potential risk factors [3]. According to the World Health Organisation (WHO), over 60% of infectious diseases in humans are zoonotic in origin, and more than 75% of newly emerging infectious diseases are transmitted from animals [4, 5]. Zoonotic diseases affect more than two billion people each year, causing around two million deaths and billions of US dollars in economic losses [6]. Over the past decade, the world has faced several major public health emergencies of zoonotic origin, including Middle East respiratory syndrome (MERS), Ebola and the COVID‐19 pandemic. Effective control of zoonotic diseases requires the integration of public health, veterinary and ecological strategies [7]. Preventing zoonotic diseases is more cost‐effective than managing outbreaks and mitigating their economic consequences, highlighting the importance of proactive measures [8]. There is increasing awareness of, and interest in, operationalizing the OH approach, supported by political and financial commitments, in order to collectively address health risks and achieve optimal health outcomes for humans, animals and ecosystems [9].

On 21 March 2014, Guinea reported a Zaire Ebola virus outbreak that spread to Liberia and Sierra Leone, becoming the largest recorded Ebola outbreak at the time, with 528 cases and 337 deaths by June 2014 [10]. A key lesson from the Ebola epidemic in Sierra Leone was the critical need for multi‐sectoral coordination and collaboration in preparing for and responding to Public Health Emergencies of International Concern (PHEIC) [11]. In response, Sierra Leone has recognized the importance of the OH approach in formalising and enhancing coordination among key ministries and agencies, including the Ministry of Health (MOH), Ministry of Agriculture and Food Security (MAFS), Ministry of Environment and Climate Change (MOECC) and the Office of National Security (ONS).

**FIGURE 1:**
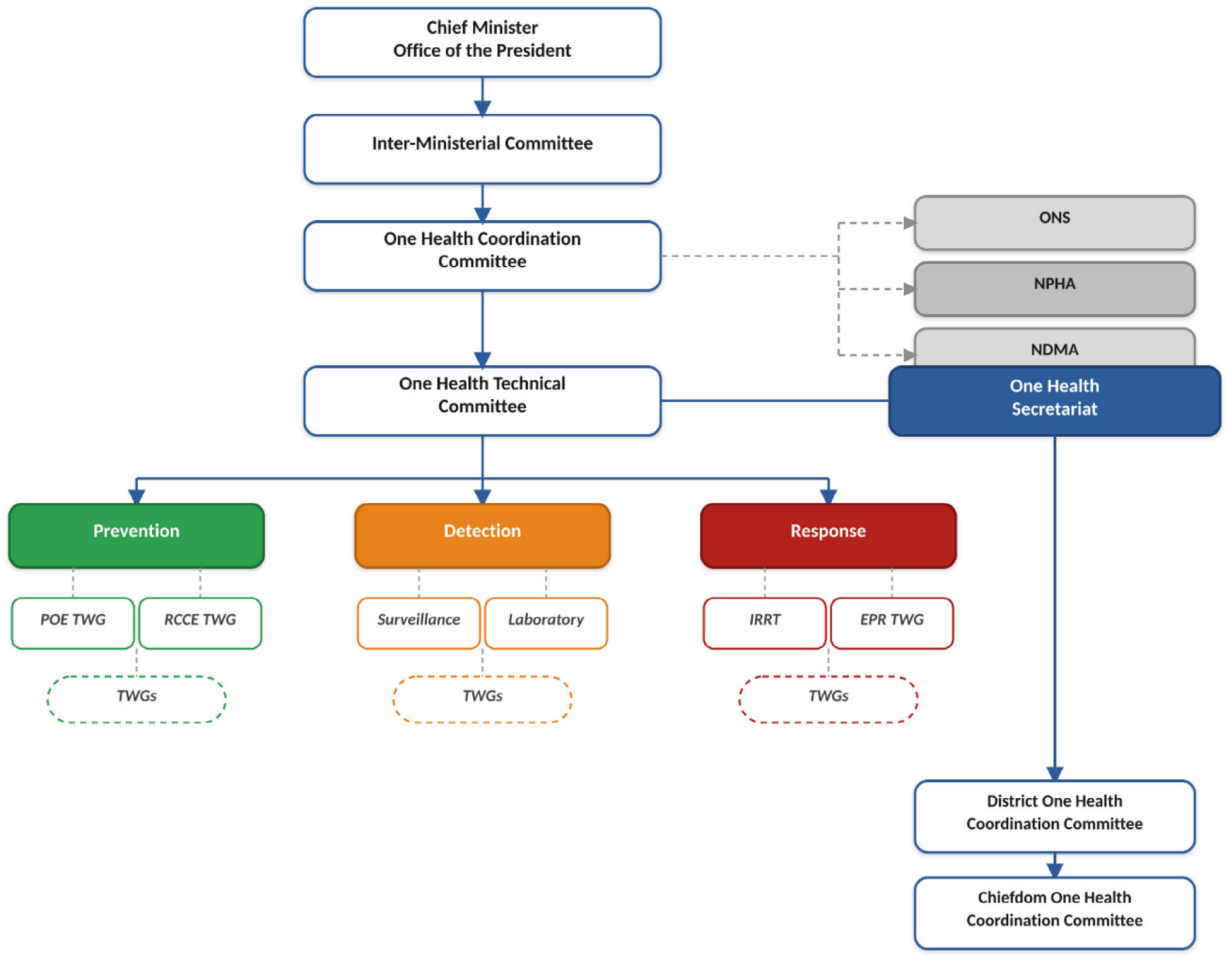
National One Health Platform of Sierra Leone. EPR, emergency preparedness and response; IRRT, integrated rapid response team; NDMA, National Disaster Management Agency; NPHA, National Public Health Agency; ONS, Office of National Security; PoE, Point of Entry; RCCE, risk communication and community engagement; TWG, technical working group.

The government's commitment to OH was demonstrated through the launch of the OH Platform in June 2017, which includes coordination committees and a dedicated Secretariat. This initiative also led to the development of key policy documents to guide OH implementation beyond the central level (Figure 1).

To date, foundational documents, such as the *National One Health Strategic Plan (NOHSP 2019–2023)* and the *One Health Governance Manual (2018)*, have been developed and were recently reviewed in 2024. However, although the OH governance framework is in place, there remains a need to operationalize the OH approach at both national and sub‐national levels, particularly in preparedness for and response to zoonotic outbreaks. This study aimed to assess the operationalization of the OH approach in two border districts, namely, Kailahun (Eastern Region) and Kambia (North–West), and to evaluate their linkage with central‐level structures in coordinating responses to zoonotic disease outbreaks. The findings are intended to inform strategies for enhancing the efficiency and effectiveness of the multi‐sectoral system in applying the OH approach to PHEIC preparedness and response.

## 2 Methods

### 2.1 Study Setting

**FIGURE 2:**
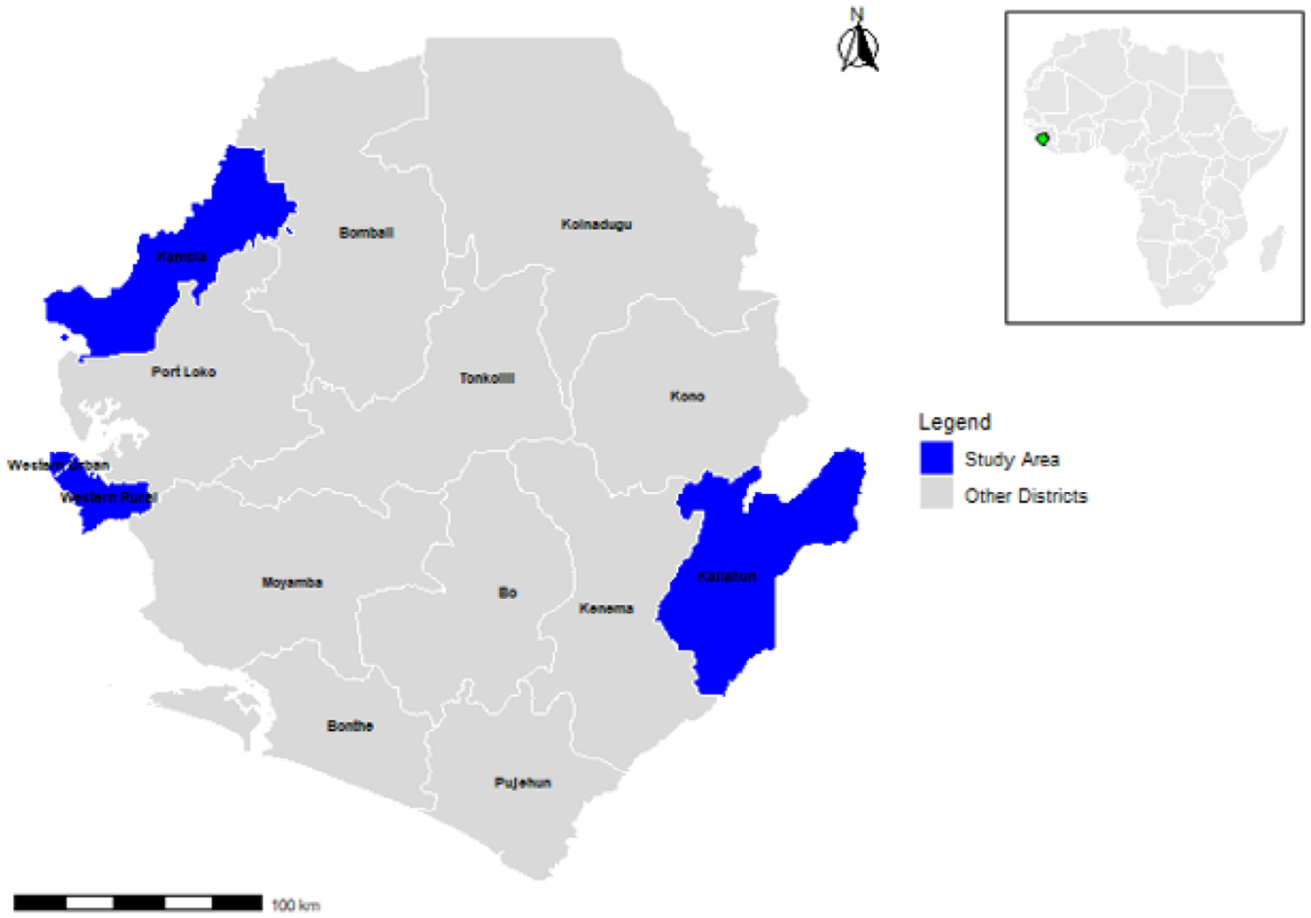
Sierra Leone map showing the study areas, including Kambia, Kailahun districts and central level (western urban and western rural area of the capital city, Freetown).

The assessment was conducted in the three geographical areas of Sierra Leone as follows: Kailahun district (east), Kambia district (North‐West) and at the central level which includes the western urban and western rural area of the capital city, Freetown (Figure 2). Both districts have open border‐crossings with replete unmanned footpaths linking Liberia and Guinea, respectively. The two neighbouring countries and Sierra Leone share a common history of the Ebola epidemic in 2014–2016 due to the shared porous boundaries. Kailahun district is more than 430 km away from Freetown (capital city) and borders Liberia to the east and Guinea to the north‐east and south (Figure 2). The district is flanked with Kenema in the south‐west and Kono in the North. Kailahun is home to twelve chiefdoms with a major international border with Guinea. Kambia district is one of the 16 districts located in the northern‐west of Sierra Leone. The district covers an area of 3108 km^2^ and has seven chiefdoms (Figure 2). The Western Area, which is divided into western rural and western urban, is the National hub for health services. With a population of approximately 1.5 million it has 110 Peripheral Health Units (PHUs) which are stratified in three categories (Maternal and Child Health Post, Community Health Posts and Community Health Centres). There are nine secondary level hospitals and four tertiary level hospitals. The administration of the health sector is primarily under the remits of the MOH at the central level and supported by the District Health Management Teams (DHMTs), which manages all health service delivery at the districts. The system is organized into three tiers: the PHU, the district hospitals and referral hospitals. The MAFS has its headquarters at the central level with offices at the districts. The MAFS has various divisions, including Livestock and Veterinary Services, Animal Production Services and the Phytosanitary unit, with mandates at the border‐crossing points. The responsibility to monitor the movement of animals into and across the country lies with the livestock assistant attached at the few functioning livestock inspection posts across the country. The MOECC with mandate to set policies for building national environment resilience in relation to climate change and natural resource management is also located at the central level. The Environmental Protection Agency (EPA) of the ECC administratively falls under the Office of the President with various subunits, including chemical control and management, field operations and extension, compliance and enforcement. Operations are mainly through the regional offices with support from the environmental officers of the district councils.

### 2.2 Study Design

#### 2.2.1 Qualitative Approach

A qualitative content analysis was used to analyse data collected through focus group (FG) interviews [12]. The manifest content, referring to the explicit and directly observable statements made by participants, was identified and interpreted at face value. Latent content, which captures the underlying meanings and interpretations behind participants’ statements, was also examined to gain deeper insights into shared experiences and perspectives. The group interactions within the FGs further enriched the interpretation of both explicit content and underlying themes. The reporting of this assessment was further guided by the Consolidated Criteria for Reporting Qualitative Studies (COREQ) [13].

#### 2.2.2 Data Collection

The qualitative interview guidelines were developed by the principal investigator (PI) with input from co‐authors. As the study was conducted in both districts, the process began with preliminary meetings held at the DHMT offices of the MOH in Kambia and Kailahun. These meetings included key district stakeholders such as the District Medical Officer (DMO), District Agricultural Officer (DAO), District Council Chairman (DCC), District Security Coordinator (DSC), representatives from the ONS and the Paramount Chief (PC) or their representative.

During these sessions, the PI briefed stakeholders on the purpose of the assessment and the data collection process involving key informant interviews (KII) and focus group discussions (FGDs). Participants were selected from among community health workers (CHWs) and members of the animal health (AH) workforce. All interviews and discussions were conducted in appropriate and accessible locations within the communities.

#### 2.2.3 Training of Data Collectors

A team of data collectors and transcribers received a 2‐day refresher training on qualitative data collection methods. The training included an orientation to the data collection tools and a refresher on transcription skills specific to qualitative research. The training was focused on data collection protocols and qualitative interview skills. Following the training, tools were later pretested to ensure appropriateness and refined on the basis of feedback from the pre‐test. Once the tools were validated, the consultant commenced data collection, starting in Kailahun district. The team then proceeded to Kambia district and later to Freetown, where data collection was completed at the central level.

### 2.3 Semi‐Structured Interviews

In‐depth interviews as guided by the semi‐structured questionnaire were conducted after consent to participate was sought (Table 1). Using the iterative approach to qualitative data collection and analysis, multiple rounds of questioning, reflecting, rephrasing, analysing and verifying after each interview to keep the focus of the assessment. Each round of interviews was used to shape the next rounds of interviews and discussions [14, 15]. The objective is to thoroughly explore every nuanced aspect of the phenomenon investigated. Information gathered from each key informant provided traceability to the data source, enabling clarification of ambiguities and resolution of information gaps. To ensure data quality, the consultant and team conducted daily debriefing sessions at the close of each day. These sessions involved reflection, field editing and verification of the consistency and security of audio recordings and field notes [16].

**TABLE 1:** Semi‐structured interview questions (KI and focus group discussion [FGD]) on One Health operationalization.

Key informant interviews (examples)	Focus group questions (examples)
1. Coordination: How do you make decisions on population health threats reported from the community? (Probes e.g.: established platform? frequency of meetings with evidence? Stakeholders involved? and challenges?)	1. How do you discuss health issues reported from the community? (Probes e.g.: meetings held with evidence? OH sectors involved? and challenges?)
2. Surveillance: How do you receive rumours or information on diseases/outbreaks from the community? (Probes e.g.: reporting tools? data‐sharing with other sectors? Reporting system? and challenges)	2. How do you get rumours/information about diseases/outbreaks from the community? (Probes e.g.: reporting tools? data‐sharing with other sectors? Reporting system? and challenges)
3. Human Resource: What is your staff capacity in addressing a range of health issues in the community? (Probes e.g.: competence? capacity‐building programme? Supervision? challenges?)	3. What can you say about the number of staff you have for the kind of work you do? (Probes e.g.: staff coverage of communities? capacity‐building programme? Supervision? challenges?)
4. Funding: Where do you get resources to support your activities? (Probes e.g.: government budget allocation? accessibility of funds? challenges)	4. Where do you get resources to support your field work? (Probes e.g.: dedicated funds? accessibility of funds? challenges?)
5. Community‐linkage: How do you involve the locals in addressing their health issues? (Probes e.g.: structures, stakeholders? challenges?)	5. How do you involve the community in your field work? (Probes e.g.: structures, stakeholders? challenges?
6. One Health Awareness: What is your knowledge of One Health? (Probes e.g.: source of knowledge? application of concept?	6. What is your knowledge of One Health? (Probes e.g.: source of knowledge? application of concept?)

### 2.4 Key Informant Interviews

The assessment explored a qualitative technique using KIIs for data collection from a range of district and central authorities that were purposely selected [17]. The selected KIs included representatives from the MOH, MAFS, EPA, ONS, District Council and Council of PCs, including the District Representatives of the for International Rescue Committee (IRC), Sierra Leone Red Cross (SLRC) and GOAL‐Sierra Leone. Participants were selected on the basis of their knowledge on the subject of interest to the assessment. The KII tool explored knowledge on nine selected themes on OH operationalization: coordination and collaboration; surveillance and reporting mechanism; human resource, capacity; communications system; funding support, community linkage and OH awareness. A total of 16 KIIs were conducted as follows: Kailahun (*n* = 7), Kambia (*n* = 6) and Central (*n* = 3). Each interview with KIs took approximately 30–45 min.

### 2.5 Focus Group Discussions

The FGD tool explored participants’ perceptions of the operationalization of OH in the context of preparedness for and response to zoonotic disease threats. The discussions were structured around the same key themes as the KII. Each FGD took approximately 45–60 min. A total of six (*n* = 6) FGDs (two per district) and two (*n* = 2) at the central level were conducted. Participants of the FGD at the district level comprise CHWs and community animal health workers (CAHWs). Both CHW and CAHW respondents represent a heterogonous mix of participants selected from various chiefdoms in Kailahun and Kambia districts. Interviews for the CHWs and CAHWs were, respectively, conducted at a convenient location of the DHMT and the MAFS offices of the districts. FGD participants at central level included staff of the MOH and MAFS. These groups of AH and human health (HH) workers were purposively selected on the basis of their direct involvement in OH initiatives and their capacity to provide insights into the operationalization of the approach. Selection of the participants from the AH sector prioritized staff of the livestock and veterinary services (epidemiology unit), including the Animal Production Unit. Participants from the HH sector included staff of the surveillance unit, Point of Entry (PoE), risk communication and community engagement (RCCE), including emergency preparedness and response (EPR). Interviews were conducted in select offices of the Public Health EOC building, the MAFS at central level, the DHMT offices in Kailahun and Kambia.

### 2.6 Data Saturation

Adequate data had been collected for detailed analysis, and no additional data could further develop or refine our themes beyond what had already been established. Throughout the data collection process, we repeatedly reviewed similar instances within the data and reached a point of saturation, where no new themes emerged. We were confident that the identified themes were fully saturated and adequately captured the breadth of the phenomenon under investigation.

### 2.7 Data Analysis

Interviews of the KIs and FGs were recorded in the digital voice recorder after participants had signed the consent form. The recordings were later transcribed verbatim, ensuring that the meaning was preserved. The transcripts were collated and analysed in a structured, multi‐phase process. The initial phase involved manifest content analysis followed by latent interpretative analysis [12]. This was done to derive underlying meanings of the phenomena. The transcripts were carefully read and reread to develop a comprehensive coding framework. Fifty codes were generated through open coding and assigned to relevant segments of the data. Thereafter, these codes were then grouped into related categories or themes, based on commonalities in the data [18]. Verbatim statements about the participants’ experiences were extracted and compiled into one text in Excel (Microsoft Corporation, USA) for each region, which constituted the unit of analysis. The data were organized according to nine predefined thematic units, including coordination, collaboration, surveillance/reporting, information‐sharing and communications, human resource, capacity, funding, community linkage and OH awareness. In a scale of 1–5, each domain was assessed within the range of ‘very weak’, ‘weak’, ‘average’, ‘strong/functional’ and ‘very strong’. For the documents review, data analysis involved extraction of narratives and descriptive data from key documents. Upon completion, this information was synthesized and aligned with the pre‐defined thematic areas [19] as shown in Table 2.

**TABLE 2:** Operational description of the elements of One Health operationalization.

Themes	Description	Sources
Coordination	Deliberate effort to engage multiple sectors, stakeholder groups and partners through a formalized structure to achieve health outcomes	‘Unified and coordinated operational structure and process that appropriately integrates all relevant stakeholders and supports the execution of core capabilities’
Collaboration	Leveraging inter‐sectorial capacity to enhance capabilities for timely detection, prevention and response to zoonotic disease threats	‘A process in which organizations exchange information, alter activities, share resources, and enhance each other's capacity for mutual benefit and a common purpose by sharing risks, responsibilities, and rewards’
Surveillance and reporting	Timely and efficient data collection, analysis and information dissemination for informed decision‐making by end‐users	‘The ability to create, maintain, support, [and] strengthen routine surveillance and detection systems and epidemiological investigation processes, as well as to expand these systems and processes in response to incidents of public health significance’
Information‐sharing and communication	Timely communication of health threats between sectors supported by a fit‐for‐purpose technologies with feedback mechanism	‘Effective intelligence/information sharing and dissemination system to provide durable, reliable and effective information exchanges (both horizontal and vertical) between those responsible for gathering information and the analysts and consumers of threat‐related information’
Human resource	The process of recruiting, training, utilizing, motivating and maintaining a skilled workforce	‘The [cap]ability to coordinate the identification, recruitment, registration, credential verification, training, and engagement of volunteers and personnel to support the jurisdictional public health agency's response to incidents of public health significance’
Capacity	The ability to perform specified functions with flexibility to accommodate and adapt vertical resources to prevent or mitigate a shared health‐threat	A combination of all the strengths and resources available within a community, society or organization that can reduce the level of risk, or the effects of a public health event (UNDRR 2017 [20])
Funding	Dedicated and accessible resource allocation by the Government for timely and effective response to health threats at the human–animal and environmental interface	‘The availability and deployment of sufficient funds at national and subnational levels to prepare for and rapidly respond to an emergency’
Community‐linkage	Connecting health care providers and the community, including public health agencies to improve access to human and animal health services which includes coordinating public health and community activities to promote healthy environment	
One Health Awareness	The knowledge of OH and the translation of that knowledge to practices that provide proof of understanding of concept	It is the collaborative, multi‐sectorial and trans‐disciplinary approach—working at the local, regional, national and global levels with the goal of achieving optimal health outcomes recognizing the interconnection between people, animals, plants and their shared environment

Abbreviation: OH, One Health.

## 3 Results

The demographic information of the KIs and respondents of the FGDs is presented in Tables 3 and 4.

**TABLE 3:** Characteristics of KIs of the study conducted in Kailahun, Kambia and central level.

District	Representative	Gender	Institution	Sector
Central	Livestock and Vet Service	Male	MAFS	Animal health
Central	Disease Surveillance	Male	MOH	Human health
Central	Chemical Controls Directorate	Male	EPA	Environmental health
Central	Border Security Directorate	Male	ONS	Security
Kailahun	District Agriculture/livestock Office	Male	MAFS	Animal health
Kailahun	DHMT Office	Male	MOH	Human health
Kailahun	District Security	Male	ONS	Security
Kailahun	IRC	Male	IRC	NGO
Kailahun	Local authority	Male	MLGRD	Local government
Kailahun	District council	Female	MLGRD	Local government
Kambia	DHMT Office	Male	MOH	Human health
Kambia	District agriculture/livestock office	Male	MAFS	Animal health
Kambia	District council	Female	MLGRD	Local government
Kambia	District environmental office	Female	MLGRD/EPA	Local government
Kambia	GOAL‐SL	Male	GOAL‐SL	NGO
Kambia	Sierra Leone Red Cross (SLRC)	Male	SLRC	NGO

Abbreviations: DHMT, District Health Management Team; EPA, Environmental Protection Agency; IRC, International Rescue Committee; MAFS, Ministry of Agriculture and Food Security; MOH, Ministry of Health; ONS, Office of National Security.

**TABLE 4:** Focused group discussion participants of Kailahun, Kambia and central level.

	FGDs with CHWs/HH staff			FGDs with CAHWs/AH staff			
Study site	Men	Women	Total	Men	Women	Total	Total participants
Central	6	2	8	6	3	9	**17**
Kailahun	10	—	10	6	2	8	**18**
Kambia	7	3	10	8	2	10	**20**
Total	23	5	28	20	7	27	**55**

Abbreviations: AH, animal health; CAHWs, community animal health workers; CHW, community health worker; FGD, focus group discussion; HH, human health.

On the basis of the pre‐determined themes, sub‐themes were generated from analysis of the content, including descriptors (supporting text quotes) which are presented in Table S1. Within the coordination theme, three sub‐themes were identified as follows: an established OH governance structure, a functional mechanism at the central level and absence of multi‐sectorial plans to undertake joint activities at sub‐national level. Within the collaboration theme, three sub‐themes were extracted as follows: meetings between sectors, clarity of roles and responsibilities and involvement of OH stakeholders unlike at sub‐national levels. Within the surveillance/reporting theme, five sub‐themes were generated as follows: existence of data‐sharing platform, conduction of joint outbreak investigations, collaboration between sectors, vertical notifications on health threats and issues of timeliness in reporting. Within the information‐sharing and communication domain, three sub‐themes were identified as follows: Information Communication Technology (ICT) infrastructure challenges, network issues limiting communicating across levels and communication of alerts. Under the human resource theme, three subthemes were elicited as follows: staff shortages, constrained supervision of staff and limited coverage of service area. Analysis of the capacity domain led to the identification of three sub‐themes as follows: the lack of skilled workforce, inadequate training, inadequate logistics and supplies. In terms of funding, four sub‐themes were extracted as follows: no dedicated resource allocation for OH, limited financial support, dependence on external assistance from partners to operationalize joint activities and lack of incentives as most CHWs (animal and human) are not under payroll. Moreover, within the community‐linkage theme, two subthemes were generated driving OH activities as follows: local relationship and informal volunteer support. Finally, four subthemes stood out prominent from our analysis of content within the OH awareness theme as follows: OH, providing a learning forum for the sectors, evidence of benefits, opportunity for resource‐sharing and OH sensitization gaps.

### 3.1 Coordination and Collaboration of OH Activities

Complex public health events involving infectious zoonotic diseases require a whole‐of‐society approach [2]. An institutionalized national coordination platform enhances coordination and collaboration among multiple stakeholders for timely detection, prevention and effective response to public health risks [21]. Our study found that at the central level in Sierra Leone, coordination and collaboration between the OH sectors is relatively strong. KIs related that there is a functional multi‐sectoral coordination mechanism, the National OH Platform (NOHP). The NOHP, which is a post‐Ebola OH initiative, was established in June 2017 to promote trans‐disciplinary approach in addressing public health threats. Our participants mentioned the platform provides a forum for effective collaboration among multidisciplinary experts beyond the health sector. These include government ministries, departments, agencies, international organizations, the private sector, civil society, media and the academia.

In the absence of an OH policy in Sierra Leone, which was yet a zero draft, the functions of the NOHP were guided by a validated governance framework, the Governance Manual of 2018. KIs mentioned that the manual specifies roles and responsibilities of the NOHP, coordination committees as well as technical working groups (TWGs) established under the NOHP. These structures were designed to streamline efforts for a seamless engagement of multidisciplinary experts in preparedness for and response to public health challenges. Another participant from our study indicated that the OH Strategic Plan (OHSP) 2016–2023 translates policy guidance from the Governance Manual to clear priority actions, timelines and budget estimates with expected health outcomes. Multi‐sectoral activities, including regular inter‐disciplinary meetings involving the animal, environment and HH sectors, are coordinated by the OH Secretariat established under the platform. Our documents review revealed that the participatory governance initiative in Sierra Leone was the result of the multi‐stakeholder collaboration gaps and lessons learnt from the early response phase of the Ebola epidemic.

By conducting the same assessment at the district level, we found a weak coordination of OH sectors. Professionals in the domain mentioned that OH governance frameworks and plans for the management of public health threats have not been adequately socialized across sectors at sub‐national level. Despite the strong OH coordination at the central level, we found no similar formal structure in either border‐crossing districts. Participants stated, OH meetings from national are formally communicated but meetings within the district are mostly informal and ad hoc. However, relative to Kambia district, collaboration between the HH and AH sectors in Kailahun has quite improved, though informal. Participants noted that the progress was primarily influenced by OH awareness among the Kailahun DHMT leadership and the voluntary role of an OH focal.

At the community level, we also found weak coordination and collaboration between the animal, environmental and HH workers in both districts. Some participants attributed the weakness to the lack of a formalized community OH structure. A shared perspective was that OH approaches were seldom implemented except occasioned by zoonotic disease threats. Another comment made was that meetings held to address emerging public health threats in the communities are mostly sector specific. The siloed approach was evidenced from the perception of respondents of FGDs held with CHWs and CAHWs in both districts. In one of the FG s held with CHWs in Kambia, Respondents said,

We the CHW system, our monthly meeting is where we are putting all of our problems that is disturbing us in our community. (FGD‐CHW, Kambia)

### 3.2 Surveillance and Reporting

Disease surveillance, which includes reporting of notifiable disease for timely decision‐making by end‐users, plays a pivotal role in early warning of infectious zoonotic disease threats. This study assesses its critical contribution in the context of OH operationalization and systems integration at national and sub‐national levels. In Sierra Leone, identifying and timely reporting of zoonotic disease threats is of national public health imperative. Our study was informed that, in 2017, guided by a tool developed by the United States Centre for Disease Control (US‐CDC), Sierra Leone conducted its first OH zoonotic disease prioritization workshop involving experts of OH sectors, including wildlife, researchers and academia. Six zoonotic diseases were ranked in order of high priority for Sierra Leone: VHFs (Viral Haemorrhagic Fevers‐Ebola, Lassa fever), Rabies, Zoonotic Influenza, Swine Salmonella, Anthrax and Plague. KIs stated, there is a functional surveillance mechanism with standard tools for reporting on notifiable Zoonotic diseases at the central level. Reporting of zoonotic health risks was not done through an institutionalized integrated platform. OH, participants related that the Integrated Disease Surveillance and Response (IDSR) strategy, though largely sectorised yet serves an opportunity for OH approach. We observed that data‐sharing between the central surveillance system and the sub‐national system was through sector‐specific reporting channels of both districts. The traditional siloed approach in reporting of health data is institutionalized over the years yet not without challenges of timeliness, completeness and validity.

Our assessment of surveillance systems at the district level found no harmonized or integrated reporting mechanism among the OH sectors. According to our district KI, a key challenge to realising a harmonized surveillance and reporting system at sub‐national level was disproportionate capacity issues in terms of skills, logistics and infrastructure, especially for the AH and environmental sector. KIs of the AH in Kambia attributed reporting weakness, in part, to lack of training in field epidemiology. OH, TWG meetings prescribed to be held regularly to enhance data‐sharing and harmonization among the OH sectors were non‐functional.

A similar perception was observed among the respondents of FGDs held in the communities of Kailahun and Kambia. Participants revealed that there was no established mechanism for data‐sharing and harmonization between the OH sectors. However, there were institutional mechanisms in place for either sector (animal or HH) for community‐based surveillance. HH respondents affirmed that internal routine meetings are followed with dissemination of reports to District Surveillance Teams via the PHU In‐Charges (a community health officer or a midwife) and the peer‐supervisors. One of the FGDs held in Kailahun district shared their reporting routines, thus;

We also have weekly reporting which are CBS – Community Base Surveillance. Every week our CHW supervisor call us to forward the information. Even when someone dies, we should call 117 to report directly. But sometimes the communication is a problem (FGD‐CHW, Kailahun)

A similar practice obtains for the AH sector in communicating surveillance updates from routine monitoring of priority animal diseases. Respondents stated that there was an existing structure in place. Indicating, surveillance reports were being shared on a weekly basis by CAHWs to the District Livestock Officers (DLOs) through the District Livestock Inspectors (DLIs). Reports collated by the DLOs/DVOs are further shared with national supervisors through the DAO. An excerpt from statements made by respondents of FGD held with CAHWs in Kambia revealed a siloed AH surveillance system. They stated thus,

Yes sir, there is a chain, that is, we have the CAHWs who report directly to the livestock assistant and, from the livestock assistant to the DLO and, then the DLO reports to the DAO and, then the DAO collates his report and forward it to the headquarters. So, this is the structure that is in place. (FGD‐CAHW, Kambia)

### 3.3 Information‐Sharing and Communications

Findings from KIs at the central level revealed a regular information‐sharing and communications between OH sectors exist. Sierra Leone has maintained its Public Health Emergency Operations Centre (PHEOC), which was established during the Ebola epidemic response between 2014 and 2016. Information‐sharing across sectors is largely delivered through the routine Emergency Preparedness and Resilient Response Group (EPRRG) coordination meetings. The EPRRG meeting, which is another post‐Ebola OH initiative, is held each Wednesday at the Public Health EOC meeting room in Cockerill, Freetown. The OHTWGs established under the NOHP serves as a precursor for interdisciplinary interface for information‐sharing and data harmonization between sectors prior to the EPRRG meetings. As at the time of this assessment, most OHTWGs were yet at their formative stage. The assessed institutional capacity of the OH sectors in terms of a functional ICT infrastructure, modern technologies and software operational skills was unequal. As such, there is an uneven commitment and participation in coordinating information and data‐sharing between sectors.

Both districts’ participants equally highlighted that there was no established communications system or platform to integrate or enhance information‐sharing among the OH sectors. A semblance of the EPRRG meetings do exist though at the sub‐national level but are specific to the institutions like the DHMT meetings of the MOH. The result of which is a weak information‐sharing among the human, environmental and AH sectors in Kambia and Kailahun districts. The absence of basic ICT equipment and internet services affects, among others, information‐sharing, collaboration and timely communication. Concerns were expressed by district KIs of recurrent issues of skills‐gap, data incompleteness and discrepancies affecting timely intervention in preventing and mitigating health threats in the communities. A KI in Kailahun said,

[Stuttering], as I told you, one of the many challenges, we don't have internet facility within the building, so we use the cafe, the cafeteria, but the second one is hmmm; our other problem is limited manpower, limited qualified personnel… (KII, MAFS, Kailahun)

We observed that the situation at the community mirrors the districts. Several participants commented that there was no reliable communication or information‐sharing system. Communications challenges were the fundamental limitations to collaboration at the local level. Communications to CHWs, including feedback on outcomes from decision‐making at the strategic level, is not done through a common OH platform. The structural and functional gaps have been largely blamed on insufficient logistics, unsustainable funding and redundant ICT equipment in district offices of both ministries (MAFS and MOH). However, participants related their local initiatives of creating social media (WhatsApp) groups as a default communications tool for CHWs, including the few CAHWs who could afford the smart phones. Highlight of some challenges mentioned by FGD respondents in Kailahun revealed thus,

By right, we are supposed to have CUG among us, which is an effective communication. Our mobiles sometimes have charging problems. We should have power banks in our hands…most of those communities except they travel five miles to charge phones and to return. (FGD, CHW, Kailahun)

**Capacity** OH implementation requires the requisite professional skills, vital trainings and necessary logistics supplies for the relevant sectors in context with the prevailing public health threats to populations and the wider environment. Our assessment found a relatively strong workforce capacity across the three traditional OH sectors (human, animal and environment sectors) at the central level of Sierra Leone. KIs stated, the relative strength was due in part, to the joint‐trainings and sector‐specific programmes targeting largely staff at the central level. There was also relative ease of interfacing and resource‐leveraging between sectors to achieve a common goal. For example, public health risk analysis and risk prioritization, which were previously HH sector‐focused, now co‐opt AH workers, including the environmental health sector. More often than the other sectors, the HH workforce has benefited from several capacity‐building efforts. These include simulations for epidemic preparedness and response, case management trainings, surveillance and cross‐border capacity‐building activities rolled out by key partners, including the USAID, WHO and US‐CDC.

On the contrary, similar assessments done at the districts revealed some observable operational capacity issues. AH sector participants underscored the operational weakness with logistics constraints in monitoring movement of animals across porous border communities. FGD respondents raised concerns over the shortage of staff with relevant veterinary skills in both districts. Our participants in the HH sector intimated that the skills‐gap and absence of environmental/wildlife officers limits OH practice. The common theme from the expressed statements across the sectors was to harmonize efforts and narrow OH skills‐gap through joint‐training programmes. For example, participants among the CAHWs mentioned about the basic training provided prior to their field deployment, such as the community‐based surveillance. Unlike the CAHWs, the CHWs had a complete training package, which comprise three different modules delivered in 21 days (in three phases) to qualify them for field work. A KI in Kailahun referenced one of such district capacity building efforts on epidemic preparedness by the GIZ.

Well, GIZ is supporting Kailahun whenever it comes to epidemic preparedness. So, through GIZ, we have been doing a lot of training of our PHU staff, on reporting on IDLcell, case definition, a lot of things. So GIZ has been supporting a lot in Kailahun for the past years with a lot of our activities, especially when it comes to Surveillance, cross‐border activities, we have been getting support from GIZ. (KI, MOH‐Kailahun)

The chiefdom level: Although previous zoonotic disease threats and outbreaks have notably been reported from the village communities, yet the capacity of CAHWs remained conspicuously suboptimal. FGD participants in both Kailahun and Kambia affirmed that there is a need for regular and structured workforce development programme and staff retention strategy for the CAHWs. A few of the respondents mentioned that training programmes for CAHWs were not followed‐up with performance evaluations to inform subsequent skills‐training programme. Apparently, the cadre of frontline workers, especially for the AH volunteers, were desirous for more trainings to meet basic expectations in terms of community surveillance and reporting. KIs at community level also affirmed the need for more training thus,

The few officers including the committed CAHWs require basic para‐veterinary service training to meet the demand of the present emerging and remerging zoonotic and Trans‐boundary Animal diseases of our time. (KII, MAF‐Central)

### 3.4 Funding 

Epidemics have far‐reaching implications beyond the health sector. We assessed the availability of funds earmarked for OH implementation in Sierra Leone. The findings show that there was no specific budget allocation for OH. We noted a common statement among KIs and respondents of FGDs across administrative levels that there were no dedicated funds at the central and district levels for OH activities. The perception was that advocacy for direct funding for OH was underexplored by the NOHP. As such, the OH Secretariat receives no national budget to coordinate interdisciplinary meetings as prescribed in the Governance Manual (2018). The OH activities are mainly supported (technical and/or financial) by developmental partners (the World Bank, USAID etc.) and/through implementing agencies or international organizations, including the WHO, World Organization for Animal Health (WOAH), Food and Agriculture Organization (FAO), German Agency for International Cooperation (GIZ) and United States Centers for Disease Prevention and Control (USCDC). Funding to implement OH gained support from partners following GoSL's expressed commitment by launching the NOHP in June 2016.

We further assessed funding for OH implementation in Kailahun and Kambia districts. KIs highlighted, there was limited motivation in operationalizing OH in Kailahun and Kambia districts with no dedicated budget allocation. The implications were that health personnel of the MOH, MAFS and the MOECC/EPA have been less incentivized to implementing OH activities in the districts. It was evident from our assessment that the absence of funds allocated to support OH operationalization was limiting institutional efforts at all levels to effectively collaborate to enhance capabilities for detection, prevention and response to public health events. One of the KIs in Kailahun said,

Actually, we don't have funds designed for One‐Health activities; we don't have funds for it. So sometimes it becomes a challenge when we have to investigate outbreaks; it's because we don't have funds. So, there is always a lot of delays related to the investigation process. (KII, MOH‐Kailahun)

### 3.5 Human Resource

Infectious zoonotic disease outbreaks sometimes demand a scaling up of the response. Regular training and maintaining a pool of human and technical resource capacity across the OH sectors is crucial to mitigating viral disease impacts on populations. We assessed the existing human resource capacity of the OH sectors. Our findings indicate that the numerical strength of staff of the AH sector was relatively weak compared to that of the HH sector. Human resource challenges were relatively more pronounced at the sub‐national levels across the three sectors. Low staff motivation was the common theme among KIs and FGs of both sectors in the districts. Due to budgetary limitations, some CHWs and CAHWs operated as volunteers (not on regular payrolls). Our study participants stated that they have to juggle between community work and menial jobs to meet basic needs.

A common theme found among participants of the districts was limited staffing of the AH sector (MAF). Some participants stated that the limited staffing of the MAF is affecting the fair distribution of CAHWs in the chiefdoms. The workforce capacity of the AH sector took a significant downturn over the years as indicated by a KI. The perception related was that the snail‐pace of recruiting new staff fell short of closing the void created through attrition. As such, there was little or no coverage by the CAHWs in some communities in Kambia. Field monitoring by the CAHWs was disproportionately restricted to the district headquarter towns with no presence at certain chiefdoms. Other participants attributed the gap as the result of government's action to retrench many trained officers who were due retirement. A KI at Central made this statement,

There is a gross shortage of human resource in the Division. Most of the trained officers were retrenched by Government order and have not been replaced up to this moment. This has contributed immensely to the quality of service and the reporting mechanism. (KII, MAF, Central)

However, among the three sectors, the environmental sector was grossly constrained in deploying staff in the districts. We observed a very thin presence of EPA/wildlife staff at district or community levels in Kambia and Kailahun. Deployment was yet limited at the provincial level. As a stopgap measure, the EPA initiated a surrogate interface with the community through the environmental officers of the District Councils.

We further analysed perceptions of FGD respondents to understand the challenges at the village community level. Views held by respondents of both the CAHWs and CHWs affirmed that operations at the community level were fraught with issues of coverage due to limited staff on payroll and weak supervision. Amidst the human resource challenges in both sectors, CHWs have relatively more presence in their catchment areas and operationally linked with their PHUs through the extended CHW programme. The AH, with limited numbers of CAHWs, was supported by few volunteers with huge coverage gap at the chiefdoms and villages. This limited coverage poses serious challenge in the AH service delivery. FGD respondents of the CAHWs expressed concerns about the need for a sustained community presence across chiefdoms and villages. Meanwhile, the environment sector had no visible presence at the village communities.

### 3.6 Community Linkage and Operations

The findings from interviews with KIs revealed an informal OH network exists between the community and the district structures, which includes the central structures (the NOHP). The AH participants in the district stated linkage with the community was largely through local authorities, farmers and other local associations of community members owning animals. Another views by participants of the HH sector were that interface with the community structures were through monthly meetings held at the PHUs, such as the Village Development Committee (VDC). A common theme among KIs was that there was no integrated approach among the OH sectors to prevent, detect or report zoonotic diseases of public health interest in the community. A similar perspective noted from a KI of the AH was that there is huge learning opportunity missed out at the community level because sectors are operating in silos. Such areas include heath‐risks associated with routine practices at slaughterhouses and slaughter‐slabs, daily cross‐border movements of animals and animal products, wet markets or holding pens at border‐crossings especially in Kambia district. Respondents further mentioned about cross‐border movement of persons and animals without screening measures in place at designated crossing points especially in Kambia district, a major economic route linking Sierra Leone and Guinea. An excerpt from an interview of a KI in Kambia highlights,

…we are having more of the animal flow from Guinea into Sierra Leone. (KII, MAFS, Kambia)

### 3.7 OH Awareness

This study assessed OH awareness among staff across the sectors, recognising that understanding health risks at the human–animal–environmental interface is critical for modifying behaviour, informing preventive actions and inspiring local response initiatives. KI interviews conducted at central level among HH, AH and EH participants revealed some disparities in the knowledge and awareness of OH. Participants (of the same sector and between sectors) do not seem to be equally informed on the OH concept, which has some influence on their perception of risks, decision‐making, practices and appreciation of OH.

At district level, besides the expressed need to collaborate across sectors, OH knowledge and awareness are even weak. There were limited outcomes to showcase as the result of OH collaboration. This is evidenced from their limited appreciation of the benefits of leveraging resources to address a shared threat.

At community levels of the districts, we found basic understanding of the concept among CHWs and CAHWs though some have had previous exposure to media information, trainings or workshops. Structural and non‐structural underlying factors limiting OH implementation at the local level could not be dissociated from the upstream factors due to the absence of policy and memorandum of understanding among the OH sectors.

## 4 Discussion

In this study, we assessed the performance of the OH approach in one urban district and two rural border‐crossing districts from other provinces in Sierra Leone. The findings indicated that coordination of OH activities was weak at both district and community levels. A similar finding was reported in Kenya, where OH implementation at the sub‐national level faced significant challenges, including sustainability issues, competing priorities and funding deficiencies [22]. Another study conducted in Nepal also reported poor coordination among governmental agencies and inadequate data‐sharing mechanisms between sectors [23]. In contrast, a study from the Netherlands reported strong OH implementation, characterized by high levels of cross‐sectoral collaboration [24].

In our study, data‐sharing mechanisms were poor at both district and community levels. The main challenges included the absence of a reporting system in the AH sector and a lack of training on OH. A study from India identified similar challenges, such as inadequate surveillance for animal diseases and poor data‐sharing mechanisms [25]. Comparable findings were reported in Oman, where surveillance of zoonotic diseases and data‐sharing resources were limited [26]. In sub‐Saharan Africa (SSA), one study highlighted those multiple platforms for data capture hindered effective information sharing and communication among OH sectors [27].

Although some level of data sharing and communication was observed in our study, there was no established communication system at either the district or community level. A study conducted in Ethiopia also identified poor integration among sectors in data sharing as a major challenge to OH implementation [28]. Our findings align with a study from Jordan, which concluded that inconsistent inter‐sectoral reporting mechanisms and data sharing for zoonotic diseases limited timely surveillance and detection of emerging and re‐emerging zoonoses [29].

Our findings also revealed that staff in the AH sector had limited competence in OH, particularly at sub‐national levels, compared to their counterparts in the HH sector. Although many online platforms, such as those provided by WOAH and WHO, offer training materials on OH, access is hindered by unreliable internet connectivity, especially in rural areas of resource‐limited countries [30, 31]. Therefore, more efforts are needed to build capacity in the AH sector. In countries with a shortage of trained veterinarians, such as Sierra Leone, training CAHWs to deliver basic veterinary services, including zoonotic disease surveillance, is essential [32]. Building OH capacity with a focus on core transdisciplinary competencies is crucial for effectively addressing disease emergencies, particularly in hotspot areas, in addition to the efforts of the national OH technical platform.

Regarding funding, our study found that there was no central budget allocated to support OH activities at the national, district or community levels. Most support came from development partners, which is not sustainable. To maintain and strengthen OH operationalization across all levels, dedicated funding through annual central budget allocations is crucial. The emerging zoonotic disease threats and cost of epidemics from infectious diseases, underscore the importance of the OH approach, especially in remote areas where human/livestock‐wildlife interactions increase the risk of pathogen spillover (Lloyd‐Smith, 2009 [33] #474;Gortazar, 2014 [34] #475;Sanchez‐Vazquez, 2021 #493). Adequate funding will enable the implementation of multidisciplinary activities that enhance the capacity to detect and respond to zoonotic diseases in a timely and effective manner [35]. The 2021 Joint External Evaluation (JEE) report indicated that zoonotic disease preparedness received more attention in SSA, where it ranked fifth among JEE sub‐areas, with SSA scoring 7% high (Elton, 2021 #494).

Another key finding of this study was the shortage of AH personnel at the community level. Chiefdoms lacked sufficient AH officers to provide basic veterinary services and coordinate OH activities. The situation was even more critical in the environmental sector, where staffing constraints at district and community levels hindered the implementation of environmental health activities. To address this, it is necessary to extend the OH concept to lower administrative levels by recruiting more CHWs, CAHWs and environmental officers, who serve as frontline workers for detecting emerging and re‐emerging diseases. Strengthening the workforce of community OH surveillance TWGs and platforms for monitoring and joint reporting is essential to expanding service coverage especially in border areas. In addition, improving working and living conditions for these staff members is important for motivation, job satisfaction and retention [36, 37, 38].

Our study also revealed a significant gap in coordination among OH sectors, particularly at the community level, which hampers the detection and reporting of zoonotic diseases. However, local community bodies in both the human and AH sectors play a vital role in linking community members with district‐level authorities. This highlights the potential of community‐based organizations to support OH activities and facilitate early disease detection. Establishing a strong community‐based surveillance system using a participatory approach can empower community members to monitor epidemics, especially in rete areas [37]. Furthermore, we observed a lack of cross‐border coordination among OH sectors at points of entry, particularly in Kambia District, which borders Guinea. A mechanism to link human and AH sectors at border points is essential for reporting public health events across jurisdictions [39, 40].

Finally, knowledge and awareness of the OH concept were not harmonized across sectors. Some central‐level staff lacked a clear understanding of the concept, and knowledge levels were even lower at district and community levels. A similar observation was made in a study conducted in neighbouring Guinea, which reported heterogeneity in knowledge across sectors regarding the definition and clinical signs of OH‐related diseases [41].

### 4.1 Limitations and Strengths

This study had several limitations. First, the short duration allocated for fieldwork may have affected the full assurance of data saturation. Moreover, there were delays in interviewing key authorities at the central level giving the structural transition of the COVID‐19 response coordination. To optimize our efforts, purposive sampling was employed, targeting only relevant individuals. Care was taken to include at least one participant from each chiefdom in the FGDs. Although conducting more FGDs per sector could have enriched the findings, the FGDs held in two distinct geographic locations (district and central levels) targeting the same sectors provided an opportunity for triangulation. Despite the narrow scope of data collection, the overall effort was considered successful.

## 5 Conclusion

The public health emergency response mechanism for zoonotic diseases of epidemic potential in Kambia and Kailahun districts, using the OH approach, was found to be weak. This was largely due to the absence of a dedicated fund to support the operationalization of OH since its inception in 2017. The lack of an earmarked funding significantly impacted the availability of essential logistics required to initiate and sustain effective coordination and collaboration among multi‐sectoral actors at sub‐national levels. As a result, critical functions such as surveillance, information sharing, communication, capacity building, human resource development and community engagement were severely constrained.

## 6 Recommendations

- **Establish and maintain functional coordination**: There is a need to establish and sustain regular OH coordination meetings at the district level, with active leadership and participation from all relevant sectors.
- **Strengthen communication systems**: To enhance OH operations across all levels, it is recommended to develop and maintain sustainable communication and information systems that link central and sub‐national structures.
- **Enhance surveillance capacity**: Actions should be taken to build upon existing district‐level AH surveillance systems to strengthen the sensitivity and responsiveness of the OH surveillance framework for early detection and reporting of emerging or re‐emerging diseases.
- **Advocate for sustainable funding:** Strong advocacy is needed to secure funding from development partners and the private sector, with a strategic plan to present a compelling case to the Government of Sierra Leone for dedicated national funding to establish a functional OH mechanism.
- **Review governance frameworks**: The OH Governance Manual should be reviewed to ensure high‐level political leadership and guidance for effective coordination of the OH platform, particularly in preparedness and response to public health emergencies.
- **Invest in training and simulation**: Regular training and simulation exercises should be conducted to build capacity in OH competencies. This will support a comprehensive approach to mitigating a range of public health risks at the human–animal–environment interface.

## Author Contributions

Joseph Anderson Bunting‐Graden conceptualized and designed the study and prepared the first draft of the manuscript. Joseph Anderson Bunting‐Graden, Alhassan Mayei, Fatmata Sawaneh, Monya Konneh, Patrick Fatoma, Emmanuel Komba Finoh, Mukeh Kenneth Fahnbulleh, Lily Kainwo, and Wesen Konteh conducted the fieldwork and performed data analysis. Foday Sahr, Rashid Ansumana, Adel Hussein Elduma Abdalla, Kinley Wangdi, Mohamed Alex Vandi, and Philip P. Mshelbwala provided technical oversight, academic supervision and critical review. All authors read and approved the final version of the manuscript. All authors contributed to the writing and finalization of the manuscript.

## Funding

The authors have nothing to report.

## Ethics Statement

Ethical approval for this study was obtained from the Njala University Institutional Review Board (IRB/NU/5/014). Additional permission to conduct the research was granted by the Sierra Leone District Health Officer (DHO).

## Consent

Informed consent was obtained from all participants prior to the commencement of the study. Literate participants provided written consent, whereas those unable to read or write gave consent via thumbprint, in the presence of a witness. To maintain confidentiality and facilitate the transcription process, each participant was assigned a unique identification number before the interviews began.

## Conflicts of Interest

The authors declare no conflicts of interest.

## Supporting information




**Supporting File**: puh270338‐sup‐0001‐Table‐5.docx

## Data Availability

The datasets generated and/or analysed during the current study are not publicly available due to restrictions imposed by the ethics approval. However, data may be made available from the corresponding author upon reasonable request, subject to institutional and ethical review.
