# Supplementary material for: Qualitative Assessment of One Health Operationalization at the Central Level and in Two Border Districts of Sierra Leone
Source: Public Health Chall. 2026 Aug 12;5(3):e70338. doi: 10.1002/puh2.70338 (PMC13465777; doi:10.1002/puh2.70338)
Supplement: Supplementary file 1 [file PUH2-5-e70338-s001.docx]

**Table 5:** Summary of themes, sub-themes and descriptors from the Qualitative Content Analysis

| Themes | Subthemes | Descriptors/Supporting Quotes |
| --- | --- | --- |
| 1. Coordination | 1.1 Governance structure 1.2 Functions 1.3 multi-sectorial Plans | - Now, there is improved coordination (EH*)\| - At the higher level, there is coordination mechanism (HH*) - We are using the OH platform to jointly communicate risks (HH*) - No plans for joint activities at the district (HH) - OH is not formally operating at Kailahun district (AH) - Coordination is poor in the district (LL) - Lack a dedicated staff to coordinate OH in the district (HH) |
| 2. Collaboration | 2.1 Meetings 2.2 Roles and responsibilities 2.3 Stakeholder involvement | - We hold OH Technical Working Group meetings (HH*) - We are implementing activities together than before (EH*) - We hold ad-hoc OH meetings (AH) - There is not much interface with the animal health (HH) - No defined roles to limit clash over activities (DSy) - Challenges in getting stakeholders to attend meetings (DSy) - In Kailahun, there is a surveillance pillar meeting in the DHMT involving MAFS (P) - This year, OH sector had met only once in Kambia (P) |
| 3. Surveillance and Reporting | 3.1 Data-sharing platform  3.2 Outbreak investigations  3.3 Collaboration  3.4 Notifications  3.5 imeliness | - We have a template for reporting animal diseases (AH) - Data from the PHUs are sent to the DHMT and later forwarded to HQ through the DHIS2 (P) - In Kailahun district we jointly hold regular surveillance meetings with animal health (HH) - We conduct joint outbreak investigations (HH) - Timeliness in reporting is a challenge due to internet issues (HH) - Sometimes we use the old reporting format because some staff more so at chiefdoms are not trained (HH) - There is no integrated reporting tool (AH) - Outbreak investigations are sometimes delayed for lack of resources (HH) - I get feedback after sending report to HQ (AH) |
| 4. Information-sharing and Communication | 4.1 ICT infrastructure  4.2 Vertical & horizontal linkage  4.3 Alerts | - Need to improve our communications and info-sharing (EH*) - We lack internet facility in the office (AH) - Network challenge is the common problem in Kailahun (P) - They use their personal phones to share updates (AH) - Severally communicate to CHWs through whatsApp forum (HH) - CHWs send information to the DHMT through in-charges (CHOs/midwifes) but not without challenges (HH) - At community level, unlike district, information sharing between sectors is informal (HH) - Communication is weak in Kambia (P) - Most times the district network coverage is erratic (DSy) - Outbreaks among animals are received late because we have limited coverage (AH) - we receive alerts from red cross volunteers (AH) |
| 5. Human Resource | 5.1 Staff shortages  5.2 Supervision  5.3 Coverage of service area | - Do not have representation in those communities (AH) - Environment has staffing limitations; we are not operational at the districts (EH) - Few CAHWs to cover the whole district (AH) - We have a number of CHWs attached to each PHU (HH) - Only have 2 staff on payroll in the district (AH) - Our problem is limited manpower and qualified officers (AH) - We don’t have control over CAHWs who are volunteers (AH) |
| 6. Capacity | 6.1 Skilled workforce 6.2 Training 6.3 Logistics & Supplies | - We are short of staff and expertise for conducting assessments and monitoring activities in the communities - We provide training for CHWs (HH) - We support the DHMT by providing targeted trainings and field logistics (P) - GIZ and FAO support in training of livestock officers (AH) - The performance of the lower cadre is below average (AH) - Data interpretation skills are lacking (P) - CHWs lack logistics to effectively do field work - I think MAFS is not as equipped as MOH (HH) - Insufficient drugs and Vets to treat animals (AH) |
| 7. Funding | 7.1 Resource allocation  7.2 Financial support  7.3 Partner assistance  7.4 Incentives | - We need the necessary resources (EH*) - Funding constraints inhibits joint activities (HH) - We don’t have funds dedicated for OH activities (HH) - Our major challenge is funding (AH) - No budget allocation for OH (AH)\| - CHWs are not on salaries (HH) - we receive logistics support for outbreaks response from Red Cross (AH) |
| 8. Community Linkage | 8.1 local-relationship  8.1 Volunteers | - We need to build our field network (EH) - we link with the communities through the CHWs - we have CAHWs in the communities and they report to the district (AH) - We depend on Red Cross community volunteers for outbreak alerts (AH - uncontrolled movement of animals across unmanned border crossings (AH) |
| 9. One Health Awareness | 9.1 Learning forum  9.2 Resource-sharing  9.3 Sensitization  9.4 Benefits | - OH enhances resource leveraging (EH*), - A platform that promotes information-sharing (HH*) - Improved knowledge of shared goals (HH*) - I have basic understanding of OH (N) \| - we realise that most of the diseases we get come from animals (PC Rep) \| - OH is a platform bringing together human and animal health (AH) - working in silos has not yielded the desired dividend (EH*) - Disparity in OH awareness and commitment by sector Heads (HH*) |

AH* = Animal Health-Central	HH* = Human health-Central	EH* = Environ Health-Central	P = Partners

AH = District Animal Health	HH = District human health	EH = District environ health	LL = Local leader

D/Sy = District Security
